# Epigenetic Mechanisms and Therapeutic Perspectives for Neurodevelopmental Disorders

**DOI:** 10.3390/ph5040369

**Published:** 2012-04-05

**Authors:** Takeo Kubota, Hirasawa Takae, Kunio Miyake

**Affiliations:** Department of Epigenetic Medicine, University of Yamanashi, 1110 Shimokato, Chuo, Yamanashi 490-3898, Japan; Email: takae@yamanashi.ac.jp (H.T.); kmiyake@yamanashi.ac.jp (K.M.)

**Keywords:** epigenetics, DNA methylation, histone modification, environmental factor, neurodevelopmental disease, reversible, drug, therapy

## Abstract

The number of children with mild neurodevelopmental disorders, such as autism, has been recently increasing in advanced countries. This increase is probably caused by environmental factors rather than genetic factors, because it is unlikely that genetic mutation rates suddenly increased within a short period. Epigenetics is a mechanism that regulates gene expression, depending not on the underlying DNA sequence but on the chemical modifications of DNA and histone proteins. Because mental stress can alter the epigenetic status in neuronal cells, environmental factors may alter brain function through epigenetic changes. However, one advantage of epigenetic changes is their reversibility. Therefore, diseases due to abnormal epigenetic regulation are theoretically treatable. In fact, several drugs for treating mental diseases are known to have restoring effects on aberrant epigenetic statuses, and a novel therapeutic strategy targeting gene has been developed. In this review, we discuss epigenetic mechanisms of congenital and acquired neurodevelopmental disorders, drugs with epigenetic effects, novel therapeutic strategies for epigenetic diseases, and future perspectives in epigenetic medicine.

## 1. Introduction

**Figure 1:**
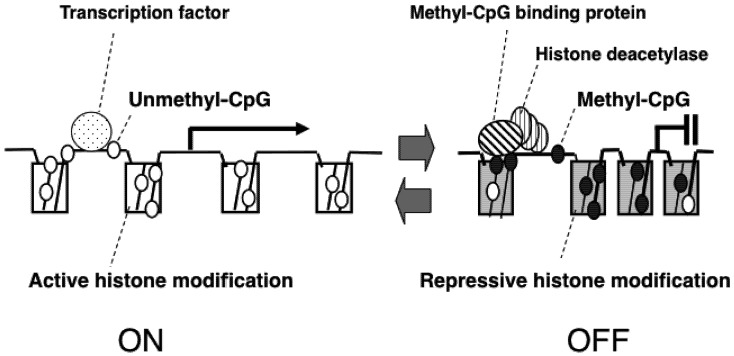
Epigenetic gene regulation via DNA methylation and histone modifications. A gene is usually activated by binding of a transcription factor to the promoter region. However, once CpG dinucleotides in this region is methylated, a methyl-CpG binding protein is bound to the methylated region, and recruits histone deacetylases. These proteins changes type of histone modification, leading to “closed” chromatin conformation, which prevents from binding of a transcription factor, leading to gene suppression.

Genomic DNA is faithfully replicated and divided between two daughter cells in the course of each cell cycle. To maintain the inheritance of gene expression patterns, the cell must not only replicate the DNA, but also duplicate its chromatin structure [1]. Following replication, DNA is methylated and packaged into nucleosomes by the binding of histone octamers to form chromatin. DNA methyltransferases (DNMTs), the enzymes that transfer methyl (CH_3_) residues to CpG dinucleotides, are coordinated with DNA replication to maintain the DNA methylation pattern (Figure 1). DNMTs recognize methylated CpG dinucleotides on the parent strand and methylate correlating CpG dinucleotides on the daughter strand [2]. This heritability of DNA methylation patterns, as well as histone modification patterns, is mediated by epigenetic machinery.

The term *epigenetics* was first used by Conrad Waddington in 1939 to describe “the causal interactions between genes and their products, which bring the phenotype into being” [3]. The current definition is “the study of heritable changes in gene expression that occur independent of changes in the primary DNA sequence” [4]. Waddington’s definition initially referred to the role of epigenetics in embryonic development, during which cells develop distinct identities despite having the same genetic information; however, the definition of epigenetics has evolved over time and is now implicated in a wide variety of biological processes, including maintenance of normal gene expression, carcinogenesis and genomic response to environmental stresses. In this review, we take a look at the current understanding of epigenetic mechanisms of neurodevelopmental disorders, and then discuss drugs with epigenetic effects, novel therapeutic strategies for epigenetic disease, and future perspectives in epigenetic medicine.

## 2. Epigenetic Mechanisms of Congenital Neurodevelopmental Disorders

Various types of genetic mutation, such as a point mutation, deletion or duplication, to a neuronal gene is known to be causes of neurological diseases. Furthermore, either no expression caused by gene deletion or over-expression caused by gene duplication can cause the same neurological phenotype in some diseases.

For example, either point mutation and deletion (both lead to no expression) or a duplication (lead to over-expression) results in similar disease phenotypes in various syndromes; mutations of the proteolipid protein 1 gene (*PLP1*) results in Pelizaeus-Merzbacher disease, a severe child onset disorder [5]; lissencephaly, a syndrome characterized by abnormal neuronal migration during fetal development, results from mutations of the lissencephaly syndrome 1 gene (*LIS1*) [6,7]; mutation of the peripheral myelin protein 22 gene (*PMP22*) causes Charcot-Marie-Tooth disease, an adult-onset neuromuscular disorder [8]; and Parkinson’s disease may results from mutation of the *a*-synuclein gene [9]. All four of these diseases are characterized by abnormal function in central or peripheral nervous system, and these indicate that almost same phenotypes can arise from either over-dosage or deficiency of certain gene product, and further suggest that the brain or nervous system is a sensitive organ to the dosages of gene products. Therefore, the proper gene regulation system is essential for the nerves system, one of which is epigenetic mechanism.

Epigenetic gene control is an intrinsic mechanism for normal tissue development and abnormalities in the molecules associated with this mechanism are known to cause various congenital diseases. It is notable that some congenital diseases with defects in epigenetic gene regulation show neurological features and mental retardation.

**Figure 2:**
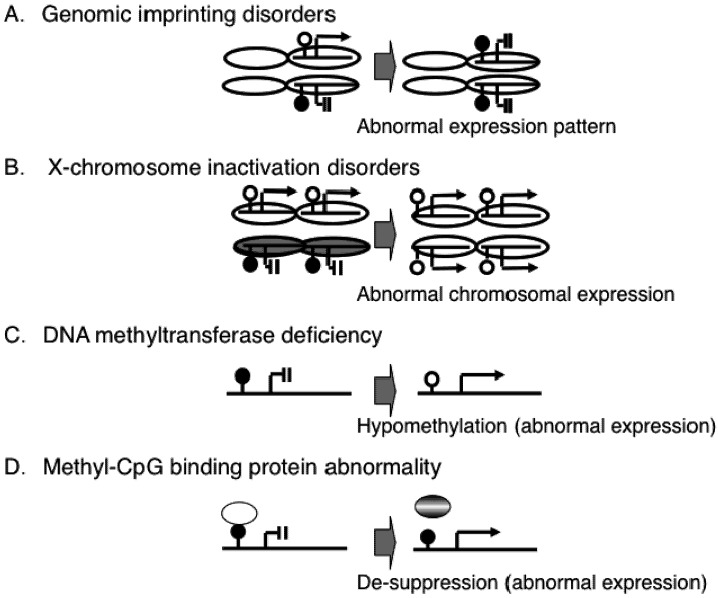
Abnormal epigenetic patterns in human congenital diseases.

Genomic imprinting is the initial epigenetic phenomenon discovered in human diseases. In an imprinted gene, one of the two parental alleles is active and the other is inactive due to an epigenetic mechanism such as DNA methylation (Figure 2A). Therefore, a defect in the active allele of the imprinted gene results in the loss of expression. This has been found in the neurodevelopmental diseases, Prader-Willi syndrome and Angelman syndrome [10].

The X chromosome has a large number of genes relative to the Y chromosome. Thus, females (XX) have more genes than males (XY). To minimize this sex imbalance, one of the two X chromosomes in females is inactivated by an epigenetic mechanism [11]. Improper X inactivation is though to be an embryonic lethal condition, which was suggested by the recent findings that majority of aborted embryonic clones produced by somatic nuclear transfer showed failure of X-chromosome inactivation [12,13], although it is difficult to directly demonstrate failure of X-chromosome inactivation in human aborted embryos.

Even when failure of X-chromosome inactivation occurs in women with one normal X chromosome and a small X chromosome due to a large terminal deletion and thus over dosage effect of X-linked genes is small, such affected women show severe congenital neurodevelopmental delays [14,15], indicating that proper epigenetic gene suppression is essential for normal development (Figure 2B).

The majority of the mammalian genome is highly methylated, with the vast majority of promoters being unmethylated. Methylation adjacent to gene promoters is actually correlated with gene expression, and non-promoter DNA methylation show more tissue-specific and developmental differences. DNA methylation occurs by the DNMT-mediated addition of a methyl group (CH_3_) to CpG dinucleotides.A defect in one of the DNMTs (e.g., DNMT3B) can cause an ICF syndrome that is characterized by immunodeficiency, centromere instability, facial abnormalities, and mild mental retardation (Figure 2C) [16,17,18]. Methyl-CpG-binding domain proteins (MBDs) are also important molecules in the control of gene expression. Mutations in one of the MBDs (e.g., MeCP2) can cause Rett syndrome, which is characterized by seizures, ataxic gait, language dysfunction, and autistic behavior [19,20]. Therefore, it is thought that MeCP2 dysfunction leads to aberrant expression of genes in the brain associated with neurological features of the disease. Recent studies have shown that MeCP2 controls a subset of neuronal genes [21,22,23,24,25], suggesting that epigenetic dysregulation of the neuronal genes may cause neurological features of the disease (Figure 2D).

## 3. Epigenetic Mechanisms of Acquired Neurodevelopmental Disorders

The Ministry of Health, Labor, and Welfare in Japan reported that the number of children with mild neurodevelopmental disorders is increasing by 10,000 cases per year [26]. Similar trends have been reported in other countries, including the US [27,28,29] and Korea [30]. The increases can be attributed, in part, to social factors, such as diagnostic substitution whereby children formerly diagnosed with mental retardation or learning disabilities are now diagnosed as being autistic. However, the increases cannot be fully explained by such diagnostic substitutions [31], and it is possible that biological changes in the brains of the children may also play a role. Thanks to advances in genomic research, several genetic factors for autism have been identified. Mutations in genes encoding synaptic molecules have been identified in a subset of autistic children [32,33]. However, the increase in autism is unlikely to be simply a result of genetic factors as there is no reason to suspect that mutation rates have suddenly increased in recent years. Rather, a more likely explanation is that environmental factors are involved in this increase. Epigenetic modifications, as well as copy number variation [34], offer one mechanism by which environmental factors might lead to changes in population health [35].

**Figure 3:**
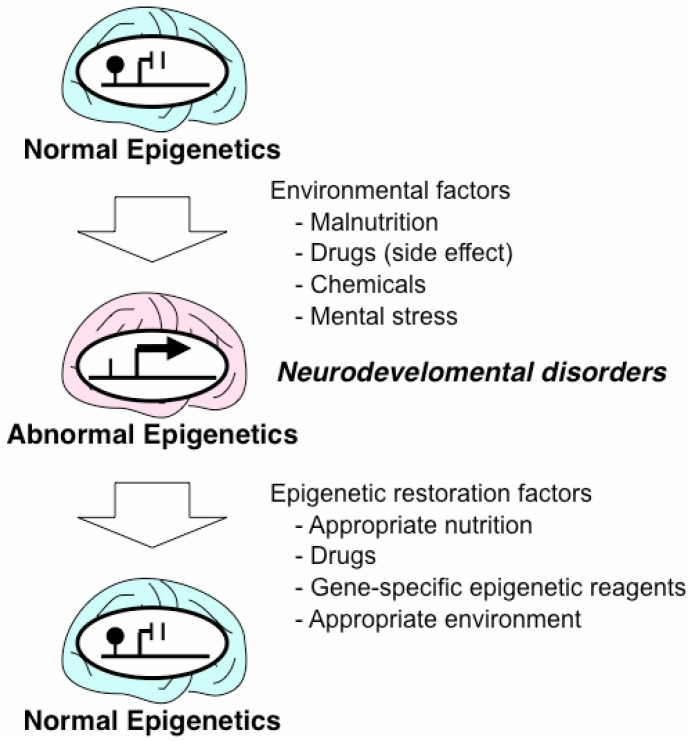
Overview of epigenetic change and environmental factors in the brain.

Short-term mental stress after birth may alter the epigenetic status in the brain and result in persistent abnormal behavior [36] (Figure 3).In rat pups from mothers exhibiting low levels of maternal care, DNA methylation at the promoter of the glucocorticoid receptor gene (*GR*), which is also known as the nuclear receptor subfamily 3 group C member 1 (*NR3C1*), increased in the hippocampus, which suppressed expression of the gene within the first week of life, whereas promoter methylation decreased in the brains of the offspring of high maternal care mothers during the same period [36]. This study was suggested to provide a putative animal model for childhood neglect and maltreatment in humans. Postmortem analysis of the hippocampus of suicide victims with a history of childhood abuse revealed the presence of hypermethylation of the neuron-specific *NR3C1* promoter in combination with a decreased level of its expression [37]. This finding suggests that the adverse effects of early-life stress on the DNA methylation programs may last throughout life [38], and also indicates that neurodevelopmental problems may arise from epigenetic dysregulation caused by environmental factors in early life (Figure 3).

## 4. Drugs and Nutrition with Epigenetic Effects

In cancer, it has been established that certain tumor types respond well to DNMT and histone deacetylase (HDAC) inhibitor treatments, with the best clinical efficacy seen in hematologic malignancies [39]. Histone methyltransferase and demethylase inhibitors are also proposed as treatments of cancer, as these enzymes are involved in tumorigenesis and tumor progression [40].

It was recently shown that the major antidepressant drug imipramine reverses the depressive state by altering an epigenetic mark (histone modification) in the brain-derived neurotrophic factor gene(*Bdnf*) in the hippocampus [41]. Similarly, the HDAC inhibitor, valproic acid, can alter the epigenetic state. Valproic acid normalizes histone acetylation of genes in the hippocampus, and suppresses of cognitive impairment by blocking aberrant neurogenesis [42]. These observations indicate that chemicals such as HDAC inhibitors that can alter epigenetic gene expression may be candidates for the treatment of neurodevelopmental and neurodegenerative diseases [43,44,45].

It is now assumed that commonly-used pharmaceutical drugs can cause persistent epigenetic changes, affecting chromatin architecture or DNA methylation, and this includes not only main effects but also side effects. Furthermore, epigenetic side-effects of pharmaceuticals may be involved in the etiology of heart disease, cancer, neurological and cognitive disorders, obesity, diabetes, infertility, and sexual dysfunction. Therefore, epigenetic assays should be incorporated into the safety assessment of pharmaceutical drugs [46]. The impact of this new approach, “pharmacoepigeneomics”, may be greater than that of pharmacogenomics [39,46].

Interestingly, epigenetic mechanisms are also involved in traditional Chinese medicine, which is a system of therapies that has developed through empiricism for over 2,100 years and is a popular alternative medicine in far east Asian countries including Japan [47]; a number of medicinal substances, used in traditional Chinese medicine, contain chemicals known to interact with epigenetic proteins, such as the Polycomb group proteins, MBD, HDAC, and DNMT [48].

Folic acid is an important nutritional factor and a mediator in the transfer of methyl groups for DNA methylation. Folic acid enters the pathway for synthesis of methionine via dihydrofolate and can be recycled through the formation of tetrahydrofolate (THF). THF is converted to 5,10-methylen-tetrahydrofolate (5,10-MTHF) and 5,10 MTHF is reduced to 5MTHF. 5MTHF then remethylates and converts homocysteine to methonine; methionine is converted to S-adenosyl-methionine (SAM), which is the major methyl donor for DNA and cellular methyltransferase reactions. Therefore, dietary folic acid may influence the maintenance of DNA methylation. Nevertheless, in contrast to the increase in US because of government mandated folic acid supplementation of grains and grain-based food products [49], folic acid deficiency during pregnancy is increasing in Japan, raising the risk of babies with neural tube defects [46]. It was recently suggested that folic acid increases histone methylation (H3K27) of the *Kdm6b* gene, decreases its expression, which leads to correction of the *Hes1* and *Neurog2* expressions that are aberrantly expressed in homogyzous *Splotch* (Sp^−/−^) mouse embryos exhibiting neural tube defects [50].

Besides epigenetic change observed in the neonatal period as described earlier, such change was also observed in the fetal period due to inappropriate supply of nutrients of the mother, which increases the susceptibility of the fetus to develop diabetes mellitus [51]. In rats, supplementing a protein-restricted diet with folic acid during pregnancy increased DNA methylation of the promoter regions of the peroxisome proliferator-activated receptor alpha gene (*Pparα* and *GR* (or *Nr3c1*) in the liver of juvenile [52] and adult offspring [53], and folic acid supplementation during the juvenile period also increased DNA methylation of the promoter regions of hepatic *Pparα*, *GR* (or *Nr3c1*), and the insulin receptor gene (*IR*), causing suppression of their expression, increased weight gain, a lower plasma b-hydroxybutyrate concentration and increased hepatic and plasma triglyceride concentrations [54]. These findings indicate that nutrient intakes may alter the phenotype of the offspring through epigenetic changes.

Since the 1980s, folic acid has been given in an empirical fashion to autistic children and to adults with mental diseases; several studies indicate that this treatment may be effective in a subset of patients [55,56,57]. As mentioned earlier, aberrant over-expression of several neuronal genes, due to the failure of epigenetic regulation, were observed in the brain of the Rett syndrome patients and that of the mouse model [21,22,23,24,25]; thus, based on the findings in the animal experiments mentioned above, it is also possible that folic acid administration may increase DNA methylation of neuronal genes and suppress their aberrant expressions in the brains of these patients.

In honeybees, female larvae fed with the secretion known as royal jelly develop as fertile queens. By contrast, their genetically identical siblings that do not receive royal jelly develop as sterile workers. Comparison of the queen and workers show that they have widespread differences in their methylation patterns. Silencing of the DNA methyltransferase gene *Dnmt3* during larval development has a royal jelly-like effect on development [58]. Moreover, the phenotypic change involved in the switch from developmental pathway of a worker bee to that of a queen can be mimicked using an siRNA that inhibits Dnmt3 [58].

## 5. Epigenetic Therapeutic Strategies for Neurodevelopmental Disorders

Epigenetic regulation is based on the attachment of modifiers, such as methyl residues, to the DNA or histone proteins; there is considerable speculation that this epigenetic mechanism can be modified in a controlled fashion by altering of the patterns of attachment and by removing such modifiers. If this proves to be the case, then epigenetically determined abnormalities in humans are in principal reversible, and therefore, potentially treatable.

Recent studies on *Mecp2* knockout mice illustrate the reversibility and treatability of neurological symptoms caused by an epigenetic failure. *Mecp2* knockout mice mimic the neurological symptoms seen in patients with Rett Syndrome, including seizures, ataxic gait, and hind-limb clasping [59]. A new Mecp2 “knock-in” mouse model was created by inserting an “exogenous” *Mecp2* gene into a Mecp2-knockout mouse [60]. To produce this phenotype, the exogenous *Mecp2* was initially silenced by an inserted stop codon, and then reactivated by treatment with tamoxifen (an estrogen analog). This treatment causes the Cre-estrogen receptor fusion protein to translocate from the cytoplasm, where it is inactive, to the nucleus, where the Cre recombinase acts to recombine the two loxP sites that flank the inserted stop codon. The mice exhibited neurological symptoms shortly after birth; however, after treatment with tamoxifen, the symptoms were much milder and the mice survived longer than first-generation Mecp2 knockout mice. These results indicate that the developmental absence of MeCP2 does not irreversibly damage neurons and that the subsequent neurological defects can be reversed. The results also indicate that neurodevelopmental disorders caused by abnormalities in epigenetic regulation, are potentially treatable after birth, and that a similar reversibility of neuronal damage might be expected not only in this DNA methylation-based epigenetic disease (Rett syndrome), but also in histone methylation based diseases [61,62,63]. Although the strategy described above (exogenous gene expression) cannot immediately be applied to human patients, because it is not possible to insert a normal *MECP2* gene into the patients prenatally, chemicals that activate the expression of *MECP2* might work in patients with Rett Syndrome, since Rett syndrome patients (all females and heterozygous) have one normal *MECP2* besides one mutant *MECP2*.

Another potential epigenetic therapeutic strategy has been developed for the imprinting disorder Angelman syndrome. In normal mice, the paternal allele of the ubiquitin-protein ligase E3A gene (*Ube3a*) is silenced and only the maternal allele is active. In a mouse model in which the maternal allele is defective (and therefore displays the equivalent mouse phenotype to Angelman syndrome), topoisomerase inhibitors have been shown to activate the silenced paternal *Ube3a* allele in neurons by reducing the transcription of an imprinted antisense RNA [64].

The nutritional supplement folic acid is generally regarded as being safe. However, there are indications that it can have a global influence on methylation rates in the genome. Possibly, it would be a better strategy with regard to epigenetic correction to target a specific gene that is associated with a disease state, rather than use treatments that have a widespread effect. One means of achieving this specific targeting may be through the use of pyrrole-imidazole (PI) polyamides. These are small synthetic molecules that recognize and attach to the minor groove of DNA, thereby inhibiting gene transcription by blocking transcription factors that bind to the DNA in a sequence-specific manner [65]. It has been shown that PI polyamide conjugated with SAHA, an HDAC inhibitor, can alter the pattern of histone modification in a gene-specific manner, resulting in up-regulation of the target gene [66].

In addition to chemical treatments, recent mouse experiments also showed that appropriate environmental conditions (e.g., providing appropriate stimuli, can ameliorate the neurological features in *Mecp2* knock-out mice by altering gene expression and synaptogenesis in the brain [67,68,69,70]. These results suggest that it might be important to provide an appropriate environment for Rett syndrome patients as this might alter their epigenetic status. On the other hand, it has been reported that an Mecp2 truncation mutant mouse (*Mecp2* ^308/+^ female mice model) was more susceptible to a specific chemical (2,2',4,4'-tetrabromodiphenyl ether 47) and showed impaired learning and long-term memory compared with a control mouse, indicating that aberrant epigenetic status leads to susceptibility to environmental factors [71].

## 6. Conclusions

The idea that characteristics acquired during an organism’s lifetime can be inherited (a concept generally associated with Jean-Baptiste Lamarck) was largely disregarded following the acceptance of Charles Darwin’s theory of natural selection as the driving force of evolution. However, the recent advances in our understanding of epigenetic inheritance may, to a limited extent, lead to the resurrection of Lamarckian inheritance. Epigenetic marks allow the transmission of gene activity states from a cell to daughter cells. Initially, it was presumed that epigenetic marks were completely erased and re-established in each generation. However, several recent studies in some model organisms indicate that the erasure process may be incomplete at some loci in the genome, and that some epigenetic marks acquired in one generation can be inherited by the next generation. This phenomenon is termed “transgenerational epigenetic inheritance” [72,73,74,75], and provides an explanation for the apparent heritability of some acquired characteristics.

The transgenerational effects of environmental toxins require either a chromosomal or epigenetic alteration in the germ line. In the rat, exposure of pregnant females to vinclozolin, an endocrine disruptor, caused infertility in some male offspring and a reduced rate of spermatogenesis in others; moreover, these effects were passed to the male offspring in subsequent generations [75]. The DNA methylation pattern in the germ line of these rats was altered from that in normal male rats, and the altered DNA methylation pattern was correlated with the effects on reproduction. Another endocrine disrupting chemical, bisphenol A, was found to alter the DNA methylation status of genes in the brain of mice during the fetal period, affecting their forebrain development [76]. Perinatal exposure to methoxychlor or estradiol benzoate has lifelong effects on neuroendocrine gene expression in rats as a consequence of changes to DNA methylation patterns [77]. Drug addiction may also be an example of a mental state influenced by epigenetic change. Cocaine and alcohol abuse were shown to alter the epigenetic state (chromatin structure) of a subset of neuronal genes, increasing the likelihood of drug addiction behavior [78,79].

One such study showed that mental stress in offspring due to maternal separation not only changed the DNA methylation status in the brain but also in the sperm, and the changed methylation status seen in the brain was accompanied by an altered expression of the corticotropin releasing factor receptor 2 gene (*Crfr2*) and altered behavior patterns [80]. These findings imply that susceptibility to mental disorders possibly be transmitted to the next generation not only as a result of specific genomic changes (mutations in genes) but also via specific epigenomic changes that are initially induced by environmental factors. In this context, future studies are necessary to establish therapeutic strategies that exploit the potential reversibility of stress-induced epigenetic modifications. Such therapeutic strategies include not only the development of epigenetic restoration drugs, but also identification of appropriate environments for maintaining a healthy state [81,82].

The methodology for epigenomic analysis has developed from the single-gene level [10] to the chromosome level [83] and now to the whole genome level [84]. Recent bead-chip technology, containing 27,000 CpG sites (updated batch contains 450,000 CpGs), has been used to identify hypomethylation at a single site of the coagulation factor II receptor-like 3 gene (*F2RL3*) in the lymphocyte genomes of heavy tobacco smokers, which might affect their future cardiovascular function [84]. Recent epigenome search identified fetus specific methylation loci on chromosome 21, in which the DNA is hypermethylated in fetuses and hypomethylated in adults [85]. Based on this finding, quantitative analysis of methylation at these loci in fetal DNA present in the maternal circulation has recently been developed to distinguish trisomy 21 (Down syndrome) and disomy 21 (normal), which will provide an accurate and noninvasive prenatal diagnostic test for trisomy 21 [86].

Understanding the human epigenome is fundamental to the investigation of congenital and acquired diseases, and will provide epigenetic biomarkers to which new drugs are targeted.
